# Radiology trainees forum survey report on workplace satisfaction, ESR education, mobility and stress level

**DOI:** 10.1007/s13244-018-0649-7

**Published:** 2018-09-05

**Authors:** European Society of Radiology (ESR)

**Affiliations:** European Society of Radiology (ESR), Am Gestade 1, 1010 Vienna, Austria

**Keywords:** Training, Workload, Job satisfaction, Workplace

## Abstract

**Objectives:**

The Radiology Trainees Forum (RTF) of the European Society of Radiology (ESR) conducted a survey among radiologists in training to gather and evaluate data on workplace satisfaction, ESR educational initiatives, mobility of professionals and stress levels.

**Methods:**

A questionnaire was forwarded to radiologists in training across Europe. The subject of the questionnaire was related to: (1) the working place, (2) safety of the working environment, (3) satisfaction at the working place, (4) familiarity with educational initiatives within the ESR and (5) reasons and motivation for mobility. Results were obtained and analysed.

**Results:**

Invitations to participate were sent by the ESR office to radiologists in training across Europe. A total of 1045 radiologists responded to the questionnaire; 77.8% were trainees and 22.2% were certified radiologists. Of the responders 65.3% considered the working place safe. Only 25.7% considered themselves involved in management, 43.9% would consider working in another country, and 52.3% were moderately satisfied with their working place. Of the responders 46.8% did not have any teaching responsibilities; 59.7% knew the European Diploma in Radiology (EDiR) and 69.7% were not aware of the content of the ESR European Training Curriculum (ETC).

**Conclusion:**

The level of training in aspects related to management safety and quality is low among trainees. The level of satisfaction at work is adequate but not sufficient. The degree of responsibility in training tasks is scarce. A small percentage is familiar with the ESR educational initiatives. As for the mobility the main reservation is lack of confidence in the training acquired.

**Main Messages:**

• *For satisfaction levels to improve, it is vital to include more creative aspects of the profession, such as research and teaching, in balance with the routine aspects of radiology.*

• *Furthermore, a greater involvement of radiologists in patient care is also essential in radiology training.*

• *To facilitate mobility, it is important to standardise training across European countries through universal programmes and training controls such as the EDiR and the ETC*.

## Introduction

Job satisfaction, safety and training currently represent important issues among radiology trainees. The European Society of Radiology’s (ESR) aim is to ensure homogeneous training across all European countries to provide the same quality of care to all European citizens as well as to promote the mobility of professionals.

The purpose of this study was to evaluate job satisfaction in radiology trainees as well as to identify factors influencing overall job satisfaction, factors that influence work satisfaction, familiarity with patient and personnel safety issues factors influencing the mobility of professionals and awareness of available ESR training programmes that can lead to harmonisation and standardisation of training.

## Materials and methods

### Design

The study was carried out as an initiative of the Radiology Trainees Forum Subcommittee of ESR through a questionnaire sent to all radiologists in training across Europe.

A total of 5291 questionnaires were sent to all radiologists in training across Europe. The survey was also posted on Facebook.

### Measures

The questionnaire is made up of five blocks of questions:

1. Working place: public, university or private practice
2. Quality, safety of the working environment and management opportunities
3. Satisfaction at the working place
4. Education, teaching, familiarity with educational initiatives within the ESR, the European Diploma in Radiology (EDiR), the European Training Curriculum (ETC - www.myESR.org/TrainingCurriculum) and the European Congress of Radiology (ECR)
5. Reasons and motivation for mobility.

### Data analysis

Statistical analysis was performed via the online tool “Survey Monkey” (SurveyMonkey Europe UC, Dublin, Ireland).

## Results

Responses were received from 1045 radiologists in training across Europe; 77.8% were trainees and 22.2% were certified radiologists, 60.0% of which had < 3 years of practice. Of the participants 87.1% were ESR members.

Of the responders 76.9% were working in university teaching hospitals. Regarding gender, 55.5% were female and 44.5% were male; 53.5% of the responders indicated not having any family responsibilities.

In the section on questions about quality, safety and management opportunities (see Table 1), 65.3% of the responders considered their work place safe; 45.3% were involved in direct patient contact. However, only 38.0% had teaching programmes in their departments on safety and quality issues related to patients; 34.5% of the responders indicated that the department provides teaching on safety and quality issues related to personnel (see Table 1).

**Table 1:** What do you think about your working environment?

I feel safe in my current work place	65.3%
I am in a capacity to influence management and professional decisions in my work place	25.7%
I work in an environment that rewards people based on their results	22.2%
I am involved in research work	38.8%
I am encouraged by my department to write scientific papers	39.7%
I am aware of my legal rights and obligations regarding my profession	50.7%
I consider that my rights and the current legislation are respected at my current job	33.5%
I am aware of the existence of a quality programme in my department	29.1%
I am involved in the development and implementation of the quality programme	15.2%
I am involved in direct patient attention	45.3%
My department provides teaching on safety and quality issues related to patients	38.0%
My department provides teaching on safety and quality issues related to personnel	34.5%

Percentages from a total of 1045 respondents (914 participants answered and 131 skipped)

In relation to management, only 25.7% considered themselves involved in management and decisions made in the working place.

In relation to working place satisfaction and stress level, 12.6% of the responders were unsatisfied in their working place, 35.1% were very satisfied and 52.3% were moderately satisfied. The majority of responders (50.6%) had working hours ranging from 35 to 50 per week.

Among the responders 64.8% considered their working place stressful to some extent and 52.6% thought they do not have enough personal time to dedicate to their family or friends. Some responders indicated that several times a week they felt exhilarated (25.5%) and emotionally drained after work (19.5%), fatigued when waking up to go to work (19.7%) and objectified patients (20.8%). However, the majority of responders felt they dealt effectively with the number of cases they were required to read daily (47.9%) and also felt they were positively influencing patient lives a few times a week (32.8%).

**Fig. 1:**
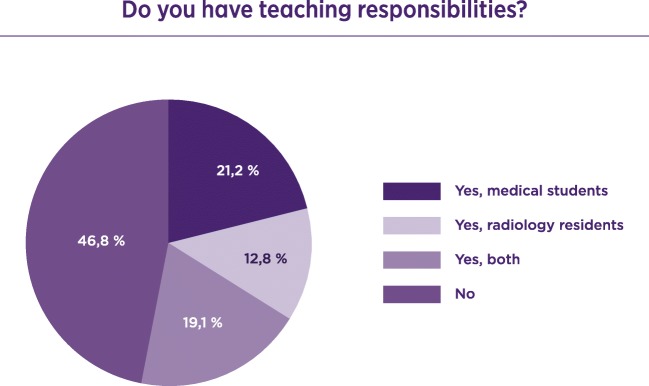
Teaching responsibilities

When considering education and teaching responsibilities among young radiologists, 46.8% of the responders did not have any teaching responsibilities in the department (Fig. 1).

**Fig. 2:**
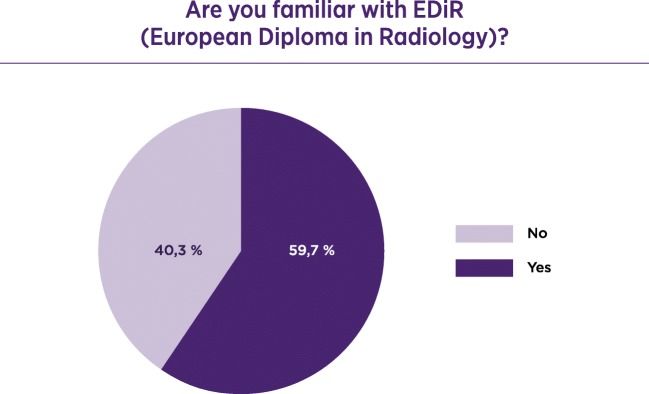
Familiarity with the European Diploma (EDiR)

Regarding familiarity with educational initiatives of the ESR, 59.7% knew the EDiR - European Diploma in Radiology (Fig. 2) of which 37.2% were made aware from the ESR website, 34.3% through colleagues, 14.0% from the ECR, 11.4% through their head of department and 3.1% from other means.

Seventy-two per cent wanted to take the EDiR, of which 66.3% felt the main reason for taking the diploma was to increase confidence in the skills acquired during their training.

**Fig. 3:**
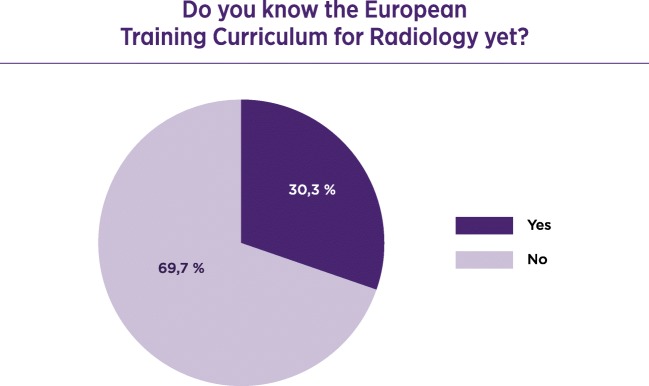
Awareness of the European Training Curriculum

Of the responders 57.8% had never attended the ECR - European Congress of Radiology; 69.7% were not aware of the content in the European Training Curriculum (Fig. 3).

**Fig. 4:**
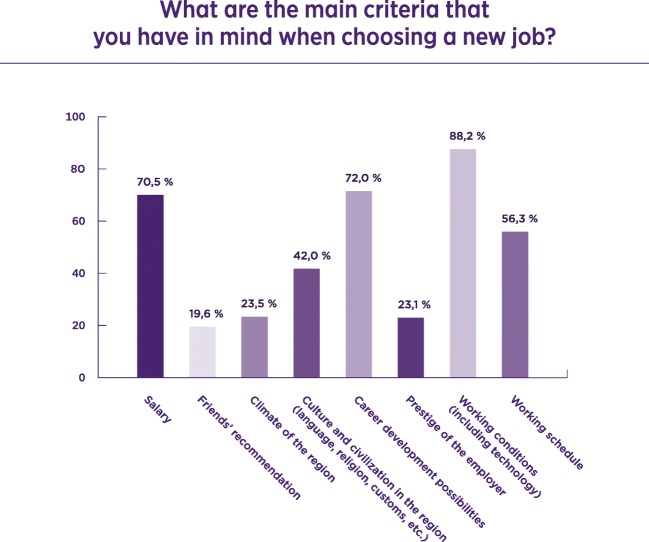
Main criteria when choosing a new job

The majority of the responders, 82.2%, were working in their native country; 43.9% would be motivated to work in another country. The reasons for working in another country include: higher income (63.6%), professional achievements (66.6%) and better working conditions (72.4%). The UK was the country where the highest percentage (57.4%) of radiologists chose as where they would like to work. The main reason for not migrating to another country is family bonds, where 52.9% of the responders strongly agreed. However a significant number of responders only moderately agreed with many of the other reasons suggested in the questionnaire. The main criteria when choosing a new job (included in Fig. 4) were the salary (70.5%), career development possibilities (72.0%) and working conditions (88.2%).

## Discussion

According to the survey the level of training in aspects related to management safety and quality needs to be worked on and improved.

It is important to take measures to emphasise the importance of providing and creating a culture of security and quality in the radiology department, implementing patient-centred care, and to facilitate training in this respect encouraging radiologists to be more active participants [1, 2].

The stress and satisfaction levels of young radiologists in their working place are satisfactory; nonetheless there is room for improvement.

A significant percentage feels only moderately satisfied (52.3%) and to some extent stressed by their work (64.8%) [3].

In terms of mobility the main reason for the lack of mobility between countries is family bonds, where 52.9% of the responders strongly agreed. An important aspect to be taken into account when establishing criteria and parameters to measure the knowledge and skills acquired during the training period is the lack of confidence in trainees of their professional skills, where 38.7% of responders moderately agreed to as a reason for not migrating to another country [4].

Overcoming this uncertainty can be achieved by using universal training programmes such as the European Diploma in Radiology (EDiR), which offers the guarantee of standardisation in acquired knowledge and ensures the confidence of trainees. A significant percentage (59.7%) of the trainees is aware of the diploma, but, on the other hand, although the diploma is based on the ESR European Training Curriculum, it is the trainees’ limited knowledge of this document that indicates that the diffusion and promotion of this information needs to be further improved [4, 5].

## Conclusion

The level of training in aspects related to management safety and quality is low among trainees and further improvement is necessary.

The level of satisfaction at work among young radiologists is adequate, but not sufficient. A high percentage of radiologists in training consider that the workplace is stressful.

The degree of responsibility and involvement in training tasks is scarce in young radiologists and a reasonable percentage is familiar with the ESR educational initiatives.

As for the mobility, it is still scarce, but a significant percentage shows interest. A relevant percentage indicates that their reservation to mobilise is lack of confidence in the training acquired.
